# Pharmacogenetic Polygenic Risk Score for Bronchodilator Response in Children and Adolescents with Asthma: Proof-of-Concept

**DOI:** 10.3390/jpm11040319

**Published:** 2021-04-20

**Authors:** Joanne E. Sordillo, Sharon M. Lutz, Michael J. McGeachie, Jessica Lasky-Su, Scott T. Weiss, Juan C. Celedón, Ann Chen Wu

**Affiliations:** 1 PRecisiOn Medicine Translational Research (PROMoTeR) Center, Department of Population Medicine, Harvard Medical School and Harvard Pilgrim Health Care Institute, Boston, MA 02215, USA; joanne_sordillo@harvardpilgrim.org (J.E.S.); sharon_lutz@harvardpilgrim.org (S.M.L.); 2 Channing Division of Network Medicine, Brigham and Women’s Hospital and Harvard Medical School, Boston, MA 02115, USA; remmg@channing.harvard.edu (M.J.M.); jessica.a.su@gmail.com (J.L.-S.); scott.weiss@channing.harvard.edu (S.T.W.); 3 Division of Pediatric Pulmonary Medicine, UPMC Children’s Hospital of Pittsburgh, University of Pittsburgh, Pittsburgh, PA 15224, USA; juan.celedon@chp.edu

**Keywords:** bronchodilator response, genome-wide interaction study, asthma

## Abstract

Genome-wide association studies (GWAS) of response to asthma medications have primarily focused on Caucasian populations, with findings that may not be generalizable to minority populations. We derived a polygenic risk score (PRS) for response to albuterol as measured by bronchodilator response (BDR), and examined the PRS in a cohort of Hispanic school-aged children with asthma. We leveraged a published GWAS of BDR to identify relevant genetic variants, and ranked the top variants according to their Combined Annotation Dependent Depletion (CADD) scores. Variants with CADD scores greater than 10 were used to compute the PRS. Once we derived the PRS, we determined the association of the PRS with BDR in a cohort of Hispanic children with asthma (the Genetics of Asthma in Costa Rica Study (GACRS)) in adjusted linear regression models. Mean BDR in GACRS participants was5.6% with a standard deviation of 10.2%. We observed a 0.63% decrease in BDR in response to albuterol for a standard deviation increase in the PRS (*p* = 0.05). We also observed decreased odds of a BDR response at or above the 12% threshold for a one standard deviation increase in the PRS (OR = 0.80 (95% CI 0.67 to 0.95)). Our findings show that combining variants from a pharmacogenetic GWAS into a PRS may be useful for predicting medication response in asthma.

## 1. Introduction

Asthma affects more than 350 million people worldwide [1], and is the most common chronic lung disease in children. Responsiveness to asthma medications varies by the individual [2], and significantly impacts quality of life for those with asthma [3]. The ability to predict patients’ responses to asthma medications will help guide their treatment and may also provide information for sub-phenotyping of asthma. While a number of genome-wide association studies (GWAS) identified individual genetic variants associated with responses to asthma medications [4,5,6,7], these single nucleotide polymorphisms (SNPs) have yet to be combined into an overall pharmacogenetic polygenic risk score (PRS) to predict response to a given medication. Furthermore, GWAS of asthma medication responses have primarily focused on Caucasian populations, with findings that may not be generalizable to minority populations. Genomic studies of asthma treatment responses will be especially important for minority populations, which are disproportionately affected by asthma. To our knowledge, no pharmacogenetic polygenic risk scores (PRSs) have been developed for an asthma treatment response.

Our aim in this proof-of-concept study was to develop a pharmacogenetic PRS for bronchodilator response (BDR) to albuterol, which is the most commonly used rescue medication for individuals with asthma. Albuterol is a short term β2 agonist that promotes bronchodilation by stimulating β2 adrenergic receptors on airway smooth muscle cells to reduce bronchoconstriction. The underlying mechanism of action for β2 agonists is mediated via increases in cyclic adenosine monophosphate (cAMP) and protein kinase A (PKA). Bronchodilator response, as an asthma phenotype, is multi-faceted, involving the epithelium [8], airway smooth muscle cells [9], and autonomic nervous system cells [10]. A number of genetic polymorphisms have been associated with BDR in GWAS.

We leveraged a GWAS of albuterol response [5] to identify the relevant GWAS variants for inclusion in our PRS. We derived a PRS for response to albuterol as measured by BDR, and tested if the PRS was associated with BDR in a cohort of Hispanic school-aged children with asthma. In addition to testing the association of BDR as a continuous outcome, we also tested BDR at two thresholds: at or above 12%, and at or above 8%. We chose to model these two cut-points as dichotomous outcomes, given that the 12% BDR threshold is often used for inclusion in asthma clinical trials [11,12], while 8% has been shown to be a both a sensitive and specific indicator of reversibility of airflow obstruction in asthma diagnosis [13].

## 2. Materials and Methods

### 2.1. Study Population, Genotypes, and BDR Phenotype

We computed the pharmacogenetic PRS for BDR in the Genetics of Asthma in Costa Rica Study (GACRS) [14,15]. GACRS is based upon a Costa Rican population from the Central Valley in Costa Rica, which is separated from the Atlantic and Pacific Oceans by mountain ranges. (Costa Rica is a Central American country located north of Panama, with coasts on the Atlantic and Pacific Oceans). The Central Valley Costa Rican population has been separated politically and geographically from other regions in Costa Rica since the initial Spanish settlement in 1569 all the way through to the late 19th century. There has been minimal immigration to the Central Valley of Costa Rica until the late 19th century, and, therefore, most of the valley’s current residents descend from approximatelyf 4000 individuals.

Participants were identified through questionnaires sent to the parents of 13,125 school-aged children (6 to 14 years) who were students at one of 113 schools in Costa Rica. These short questionnaires were sent out by mail from February 2001 to December 2006. Of the approximately 55% of children whose parents returned the questionnaire (N = 7282), 37% (N = 2714) had asthma. Asthma was defined as physician-diagnosed asthma and at least two respiratory symptoms or asthma attacks in the previous year. Participants who had a high probability of having at least six great-grandparents born in the Central Valley of Costa Rica and were willing to participate were enrolled in our study. Of these participants, 967 had genotyping, lung function assessments, and relevant covariates, and were included in our data analysis. There was no significant difference in sex or grade in school between children who did and did not agree to participate in the study. The majority of children in GACRS had mild to moderate asthma, and 51% were on inhaled steroids.

Genotyping was conducted using the Illumina Bead Station 500G system by Illumina Inc. (San Diego, CA, USA). BDR to inhaled albuterol was calculated as the percent change in forced expiratory volume in one second (FEV_1_): BDR = 100 × ((postFEV_1_ − preFEV_1_)/preFEV1)), where preFEV_1_ is the lung function before albuterol treatment (baseline) and postFEV_1_ is the lung function following albuterol treatment.

### 2.2. Statistical Analyses

For PRS computation, we used results from a GWAS of BDR in a Caucasian cohort from Himes et al. [5]. This GWAS included multiple asthma clinical trials: CAMP (the Child Asthma Management Program, a clinical trial to evaluate the long term effects of budesonide and nedocromil on mild to moderate asthma in children) [16], Leukotriene Modifier, or Corticosteroid Salmeterol study (LOCCS) in which participants had well-controlled asthma on inhaled corticosteroids (ICS) [17], Effectiveness of Low Dose Theophylline as an Add-on Treatment in Asthma trial (LODO) in individuals with poorly controlled asthma [18], a medication trial in individuals with moderate to severe asthma conducted by Sepracor, Inc, [19,20], and subsets of clinical trials within the Childhood Asthma Research and Education (CARE) network including individuals with mild to moderate asthma [21], and the Asthma Clinical Research Network (ACRN) participating in the NHLBI SNP Health Association Resource (SHARe) Asthma Resource project (SHARP), which included individuals with a range of asthma severity (mild to severely persistent) [22]. We ranked the top GWAS polymorphisms (*p* < 0.001) by Combined Annotation Dependent Depletion (CADD) scores. CADD scores integrate multiple annotations into one score, in a way that prioritizes functional, deleterious, and disease causal variants across a wide range of functional categories [23,24]. We used SNP-Nexus (https://www.snp-nexus.org/v4/, accessed on 12 January 2020) to annotate the SNPs with CADD scores (specifically, the PHRED-like scaled CADD score) [25]. CADD scores are derived using a machine learning algorithm that is trained on a binary distinction between simulated de novo variants and variants that have arisen and become fixed in human populations, since evolutionary branching between humans and chimpanzees occurred several million years ago. Simulated de novo variants may include both neutral and deleterious alleles, given that they are free of selective pressure. Variants that have become fixed in human populations since the split between humans and chimpanzees are most likely neutral or weakly deleterious, as they have survived millions of years of selection [24].

Of the polymorphisms at *p* < 0.001, 16 SNPs had a CADD score of 10 or higher. A CADD score of 10 indicates that these polymorphisms are predicted to be within the top 10% most deleterious substitutions that can occur in the human genome. We extracted genotyping data on these 16 polymorphisms for GACRS participants. We created a PRS for each participant using the genetic effect sizes (i.e., beta values published in Himes et al. [5]) as weights. In our PRS calculation, we accounted for the number of effect alleles an individual has. The PRS was calculated as follows using genotype data for *m* SNPs, based on estimated SNP effect sizes (*β_J_*) from GWAS summary data.

(1) $$PRS_{i}=\overset{m}{\underset{j=1}{\sum }}x_{ij}\hat{\beta_{J}}$$

where *x_ij_* is the genotype for the *i*th individual and the *j*th SNP, encoded as 0, 1, or 2 minor alleles. Using linear regression models adjusted for age, sex, BMI category, genotype PCs 1–6, and doctor visits for asthma in the past year, we determined the association between a standard deviation increase in the PRS and percent of BDR. We tested sex and body mass index (BMI) for an interaction with the PRS on BDR in our linear regression models. We also determined the association between the PRS and odds of having a BDR of 12% or higher, in models adjusting for age, sex, BMI category, genotype principal components, and doctor visits for asthma. We chose to use a 12% cut-point for BDR, given that National Asthma Education and Prevention Program’s Expert Panel Report uses the 12% cutoff as evidence of airway reversibility in establishing the diagnosis of asthma [26]. The 12% cut-point for BDR is also often used as an inclusion criterion for asthma clinical trials as well [11,12]. We conducted a sensitivity analysis with a cut-point of 8% (considering those at or above 8% as “responders” vs. those below 8% as “non-responders”). We performed this sensitivity analysis with the 8% cut point, as prior work has demonstrated that this threshold may be a more sensitive and specific classifier of airway reversibility in asthma [13]. To visually display connections between genes used in the PRS calculation and relevant proteins, we constructed a network with our PRS genes as input using an online network analysis tool (https://www.networkanalyst.ca/NetworkAnalyst/home.xhtml, accessed on 5 April 2021).

## 3. Results

**Figure 1:**
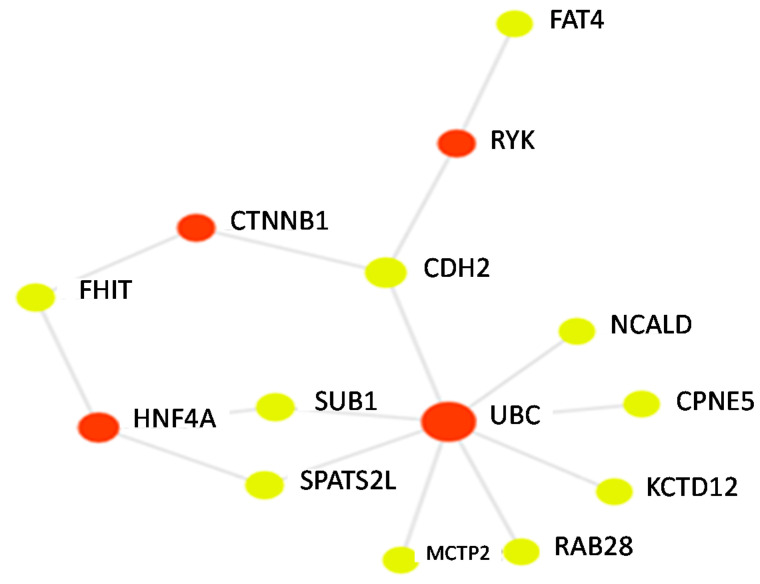
A gene protein network connecting seed genes (yellow nodes) represented in the PRS with each other and with relevant proteins (red-nodes).

GACRS participants had a mean of 9.3 years of age (standard deviation of 1.9 years) and 59% percent were male. With respect to BMI classification, 65% of participants were normal, 3% were underweight, 15% were overweight, and 17% were obese. BDR in GACRS participants had a mean of 5.6% with a standard deviation of 10.2%. Participants had a median of 3 doctor visits per year (interquartile range was 1 to 5 doctor visits for asthma). The observed BDR in GACRS is somewhat lower than the BDR levels often observed in asthma clinical trial participants. All 16 of the top SNPs (*p* < 0.001) with CADD scores above ten from the Himes et al. primary GWAS of BDR had been genotyped or imputed in our GACRS data. For these 16 SNPs, we used the weights (i.e., Betas) from the Himes et al. study [5] to derive our pharmacogenetic PRS (Table 1). SNPs in the following genes were included in the PRS calculation: *TMEM51* (transmembrane protein 51), *LMX1A* (LIM homeobox transcription factor 1 alpha), *SLC2A13* (solute carrier family 2 member 13), *KCTD12* (potassium channel tetramerization domain containing 12), *SLITRK5* (SLIT and NTRK like family member 5), *MCTP2* (multiple C2 and transmembrane domain containing 2), *CDH2* (cadherin 2), *SPATS2L* (spermatogenesis associated serine rich 2 like protein), *FHIT* (fragile histidine triad diadenosine triphosphatase), *FAT4* (FAT atypical cadherin 4), *RAB28* (RAB28 member RAS oncogene family), *SUB1* (SUB1 regulator of transcription), *CPNE5* (copine 5), *NCALD* (neurocalcin delta), and *FRMPD1* (FERM and PDZ domain containing 1). A visualization of PRS genes and connected proteins is shown in Figure 1. Of the genes represented in Table 1, 10 were connected in a gene/protein network. Multiple genes (*NCALD*, *CPNE5*, *KCTD12*, *RAB28*, *MCTP2*, *SPATS2L*, *SUB1*, and *CDH2*) were connected to UBC (ubiquitin C). *SPATS2L* and *SUB1* were also connected to *HNF4* (hepatocyte nuclear factor 4 alpha). Other genes were connected to RYK (receptor-like tyrosine kinase), and CTNNB1 (catenin beta 1). The PRS we derived was normally distributed, with a mean of 3.84 and a standard deviation of 7.41. In the linear regression models adjusted for age, BMI, sex, the first six principal components of genotype, and doctor visits for asthma, we observed a 0.63% decrease in BDR for a standard deviation increase in the PRS (*p* = 0.05). We did not observe an interaction between the PRS and sex or age on BDR (*p* > 0.10).

We also tested our PRS for BDR in logistic regression models with 12% and 8% BDR thresholds as dichotomous outcomes. In GACRS, 166 participants (17%) had a BDR of 12% or higher, while 275 participants (28.4%) had a BDR of 8% or higher. In models adjusted for age, BMI, sex, genotype PCs 1–6, and doctor visits for asthma, we observed an odds ratio of 0.80 (95% CI 0.67 to 0.95) for BDR of 12% or higher with a standard deviation increase in the PRS. In our sensitivity analyses using a BDR of at least 8% as the outcome (adjusted for the same set of covariates) we observed, an odds ratio of 0.80 (95% CI 0.69 to 0.92) for a standard deviation increase in the PRS for BDR.

## 4. Discussion

In this proof-of concept study, we leveraged summary statistics from a large GWAS of BDR to derive a pharmacogenetic PRS for response to albuterol in an independent population. Our PRS for BDR was associated both with a continuous BDR response as well as BDR at or above two clinically meaningful thresholds (12% and 8%). While genetic association studies of response to medications have often uncovered individual variants with large effect sizes [27], GWAS of medication responses in asthma (i.e., for inhaled corticosteroids and albuterol) have uncovered multiple variants with small effect sizes. Creating an index that combines all of these genetic variants into a single score may be a more powerful predictor of asthma medication response than any of the individual variants alone, with enhanced utility for precision medicine applications. One of the main challenges in PRS methodology is the determination of SNPs for inclusion in the score. Rather than focus on *p*-values alone for SNP selection, we chose to incorporate data on the potential functional relevance of SNPs when identifying variants to include in the PRS.

We selected GWAS variants for inclusion in the PRS based on CADD score annotated results from a Caucasian cohort, and tested our PRS for BDR in a Hispanic cohort. CADD scores correlate with experimentally measured regulatory effects, known pathogenic variants and complex trait associations, and are useful filtering criteria for capturing variants in genes with functional relevance [23]. Genes represented within our CADD score’s derived PRS have potential biological connections with our BDR outcome. For instance, our CADD score derived PRS includes a variant within the *SLITRK5* gene, which is known to encode for neurotrophin receptors. Recent studies show expression of neurotrophins and their receptors in airway smooth muscle [28], and have also identified a role of these mediators in contractility of the airways [29]. A variant near *CDH2* was also included in our PRS calculation. *CDH2* encodes for N-cadherin, which is a transmembrane protein known to have reduced expression in the airway epithelium of patients with asthma [30]. *FAT2*, also included in our PRS score, is a gene that encodes for proto-cadherin. Lastly, a variant in *SPATS2L*, the gene that was the primary focus of the discovery GWAS on which our PRS study is based, also emerged as important using the CADD score ranking of GWAS results. An in vitro *SPATS2L* knock down study in airway smooth muscle cells (conducted as part of Himes et al.) revealed a potential functional role of *SPATS2L* in down-regulating the number of β2-adrenergic receptors [5]. Of note, the *SPATS2L* variant included in our CADD score-based PRS (rs3739118) is different from the top *SPATS2L* variant identified in the Himes et al. GWAS (rs295137). These two SNPs are not in linkage disequilibrium with one another (R^2^ = 0.40), and the former is an intron variant, while the latter (rs295137) is near but not located within the *SPATS2L* gene.

Strengths of our study include the prioritization of SNPs with potential functional relevance in our PRS calculation. Inclusion of minority participants in our analysis of a PRS for the asthma medication response is also a major strength of our study. Minority patients generally receive poorer quality asthma care than white Americans, even after adjusting for income and insurance status [16]. Reducing racial disparities in asthma clinical care requires an understanding of how genetics plays a role in medication responses [31,32]. Our study demonstrates the feasibility of a PRS for BDR in a Hispanic population (Costa Ricans) that are disproportionately affected by asthma. Weaknesses of the present analysis are that only two populations (a published GWAS to identify PRS polymorphisms and testing of the PRS in GACRS) informed this study and that the sample sizes may be small for establishing a PRS. Future work will incorporate the testing of our PRS for BDR in additional populations. Given that our PRS for BDR was derived based on a GWAS that included both children and adults, it will be interesting to test whether it also performs well in adult populations. We tested our PRS only in children (elementary school-aged children and early adolescents) in the present study. We should note that, while we did observe a statistically significant association for the PRS and BDR as a continuous outcome, the overall effect size was relatively small. When considering a threshold of BDR response at clinically meaningful values (≥12% or ≥8%), the PRS was associated with a decreased odds bronchodilator responsiveness. The threshold model may be more meaningful for clinical use and interpretation.

In conclusion, this proof-of-concept study demonstrates that developing pharmacogenetic PRSs could aid precision medicine efforts in asthma care. The use of SNP prioritization through functional annotation (i.e., CADD scores) is an important new approach to consider for PRSs of pharmacogenetic treatment responses, and may have implications for precision medicine efforts beyond asthma.

## Figures and Tables

**Table 1:** SNPs included in the polygenic risk score (PRS) calculation for the bronchodilator response (BDR) (with Betas from Himes et al.).

Chr	bp	rsID	Effect Allele	Beta	Gene/Nearest Gene
1	15539953	rs1316277	T	−3.51	*TMEM51*
1	165140258	rs7540787	T	−2.70	*LMX1A*
12	40441521	rs10878035	C	4.85	*SLC2A13*
13	77062997	rs581121	T	3.17	*KCTD12*
13	89392881	rs4270027	G	−3.30	*SLITRK5*
14	86791228	rs1505187	A	3.63	
15	95588003	rs8031253	T	2.98	*MCTP2*
18	25760028	rs11564299	G	3.44	*CDH2*
2	201253769	rs3739118	A	2.81	*SPATS2L*
3	60531242	rs6807877	A	5.36	*FHIT*
4	126967979	rs6534528	G	−3.77	*FAT4*
4	12935474	rs6811193	G	−4.23	*RAB28*
5	32581603	rs6876035	T	2.58	*SUB1*
6	36772543	rs9296197	G	3.95	*CPNE5*
8	103190097	rs1265121	T	2.80	*NCALD*
9	37666010	rs2057643	C	3.50	*FRMPD1*

## Data Availability

The data presented in this study are openly available in dbGaP (https://www.ncbi.nlm.nih.gov/gap/, accessed on 5 April 2021) accession no. phs000988.v4.p1.
